# Evaluation of orthodontists’ attitudes and practices regarding residual resin removal methods

**DOI:** 10.1590/2177-6709.29.3.e242402.oar

**Published:** 2024-07-08

**Authors:** Lívia Lima de Moraes BARRETO, Sarah Aquino de ALMEIDA, Fernanda Campos MACHADO, Robert Willer Farinazzo VITRAL, Marcio José da Silva CAMPOS

**Affiliations:** 1 Federal University of Juiz de Fora, Department of Orthodontics (Juiz de Fora/MG, Brazil).

**Keywords:** Composite resin, Removal, Orthodontics, Questionnaire, Resina composta, Remoção, Ortodontia, Questionário

## Abstract

**Introduction:**

The removal of residual resins is a routine procedure in orthodontic clinics and of great importance to the final result of the treatment.

**Objective:**

To evaluate the main methods of residual resin removal used by orthodontists, and the main reasons for choosing these methods.

**Methods:**

A questionnaire consisting of 21 questions: 6 relating to demographic data and the other 15 relating to two methods used to remove residual resins (drills or pliers) was sent by e-mail to orthodontists registered with the Regional Councils of Dentistry of São Paulo and Rio de Janeiro (Brazil) within April and June, 2023. Questionnaires were sent back by 153 professionals.

**Results:**

Residual resin removal is always carried out with high speed drill for 44.7% of the sample, and with low speed drill for 28.7%; 61.3% use irrigation. The multi-laminate carbide bur is used by 82.5% of orthodontists. Pliers are always used by 12.4%. Resin-removing pliers with Widia are used in 39% of cases. The use of high speed was justified by the shorter working time, and the choice of pliers was justified by the smaller damage to the tooth enamel.

**Conclusion:**

The most used residual resin removal method was the multi-laminate carbide bur at high speed with irrigation, justified the by shorter working time.

## RESUMO

**Introdução:**

A remoção de resinas residuais é um procedimento rotineiro na clínica ortodôntica e de grande importância para o resultado final do tratamento.

**Objetivo:**

Avaliar os principais métodos para remoção de resinas residuais utilizados por ortodontistas e os principais motivos para a escolha desses métodos.

**Métodos:**

Um questionário composto por 21 perguntas: 6 relativas aos dados demográficos e 15 relativas aos métodos de remoção de resinas residuais (brocas ou alicates) foi enviado por e-mail para ortodontistas dos conselhos regionais de São Paulo e Rio de Janeiro entre os meses de abril e junho de 2023, com o total de 153 respostas.

**Resultados:**

A remoção de resinas residuais é realizada sempre com brocas em alta rotação para 44,7% da amostra e em baixa rotação para 28,7%; 61,3% utilizam irrigação. A broca carbide multilaminada é utilizada por 82,5% dos ortodontistas. Os alicates são sempre utilizados por 12,4%. O alicate removedor de resina com Wídea é utilizado em 39% dos casos. O uso da alta rotação foi justificado pelo menor tempo de trabalho e a escolha por alicates, justificada pelos menores danos ao esmalte.

**Conclusão:**

O método de remoção de resinas residual mais utilizado foi a broca carbide multilaminada em alta rotação com irrigação, justificado pelo menor tempo de trabalho.

## INTRODUCTION

Orthodontic practice underwent a major impact after the introduction of composite adhesion to tooth surface.^1^ Treatments that used brackets welded to bands on all teeth were replaced by accessories bonded directly to the teeth.^2^ The bonding using restorative material on the tooth surface was introduced in dentistry in 1955 by Buonocore^3^ and later, in 1965, Newman^4^ made metallic material possible to be bonded to the enamel surface.^5^ Since then, adhesion has been used in orthodontic clinics for direct bonding of brackets or making attachments, whether in treatment with full fixed appliances or aligners.

Optimal adhesion in Orthodontics should offer sufficient strength to withstand masticatory and orthodontic forces, and at the same time allow easy and safe removal of the devices used for tooth movement, avoiding permanent damage to tooth enamel and/or the persistence of material residues after debonding procedures.^6^ ^-^ ^8^

Different methods for removing residual resins are used, in order to remove the remnants without damaging the underlying enamel^8^, such as: manual removal methods using pliers, tungsten carbide or composite burs mounted on low or high speed handpieces^8^, with or without the use of irrigation^9^, in addition to the possibility of using high-power lasers.^10^

However, even though the bonding systems have evolved and the methods for removing residual resins have varied widely, all techniques end up causing some damage to the enamel surface.^7^ ^,^ ^8^ ^,^ ^11^ This is frequent because the hardness of the materials most commonly used to remove the composite (quartz, aluminum, steel, carbon, zirconium oxide and tungsten carbide) is higher than the hardness of the enamel,^7^ making it difficult to establish protocols for removing residual resins.^8^

Considering the importance of bonding in Orthodontics, as well as the removal of residual resins, this study aimed to find out the most common attitudes and practices used by orthodontists in their day-to-day work to remove bonding resins remnants.

## MATERIAL AND METHODS

This study was approved by the Research Ethics Committee of the Federal University of Juiz de Fora under protocol number 5.658.797, and all participants signed an informed consent form.

The first version of the questionnaire was sent via link to 47 orthodontists in the state of Rio de Janeiro (Brazil), using orthodontic messaging groups, being accompanied by a text presenting the study and the informed consent form. Then the instruments and the most common practices used were selected to elaborate the final version of the questionnaire.

Afterwards, ten experts answered the questionnaire at T0 and ten days later (Test-Retest), to calibrate the instrument. The format of the tested questionnaire was kept unchanged.

The final version of the questionnaire consisted of 21 questions: 6 relating to demographic data and the other 15 relating to residual resin removal methods (Table 1). It was sent by e-mail to orthodontists registered with the Regional Councils of Dentistry of São Paulo and Rio de Janeiro (Brazil), accompanied by a text presenting the research and the informed consent form, within April and June, 2023. At the end of this period, 153 responses were collected, and the sample number was calculated using the chi-square test, with a power of 83% and an alpha error of 5%.

**Table 1:** Final questionnaire: questions relating to residual resin removal methods.

QUESTIONS	
1.	What type of orthodontic appliances do you use to carry out orthodontic treatments?
2.	Do you use high speed drill to remove residual resin from bonding in Orthodontics?
3.	Do you use irrigation during the procedure of removing residual resins with high speed drill?
4.	Do you use low speed drill to remove residual resin from bonding in Orthodontics?
5.	Do you use pliers to remove residual resins from bonding in Orthodontics?
6.	Specify one or more type of burs that you use to remove residual resin in Orthodontics.
7.	Which pliers do you use to remove residual resins in Orthodontics?
8.	Specify one or more reasons why you use high or low speed drill to remove residual resin in Orthodontics.
9.	Specify one or more reasons why you use pliers to remove residual resin from bonding in Orthodontics.
10.	Specify one or more reasons why you DO NOT use high or low speed drill to remove residual resin in Orthodontics.
11.	Specify one or more reasons why you DO NOT use pliers to remove residual resin in Orthodontics.
12.	Specify one or more reasons why you DO NOT use irrigation to remove residual resins with high-speed drill.
13.	Does the use of HIGH SPEED drill WITHOUT IRRIGATION to remove residual resins cause PAIN/DISCOMFORT for the patient?
14.	Do you use another method of removing residual resins other than those mentioned?
15.	If you do use more than one TECHNIQUE to remove residual resins, what factors do you evaluate to make a decision about which technique to use?

The answers were presented in tables with their respective absolute (n) and relative (%) frequencies. The McNemar-Bowker test for paired nominal data was applied in the test-retest phase. The Cronbach’s alpha coefficient (α) was applied to test the degree of reliability of the instrument. Pearson’s chi-square test was used to compare the differences associated with the variables ‘gender’ and ‘type of graduation’. For all the measures, an alpha value was assumed to be significant for *p*<0.05. The analyses were carried out in STATA v. 15 (Data Analysis and Statistical Software College Station, Texas, USA).

## RESULTS

The analysis of the answers given by the ten participants in the test (T0) and retest (10 days later) showed a *p*> 0.05 for all the variables. Moreover, there was no significant difference in the pattern of answers at the two moments evaluated. Therefore, the instrument was considered calibrated. Cronbach’s alpha was 0.827, indicating that the instrument was accurate and reliable to evaluate the residual resin removal practices.^12^

The sample was formed by 153 orthodontists from the Brazilian Southeast states of São Paulo (66%) and Rio de Janeiro (44%). The male representation in the sample was 36.6%, at an average age of 49.2 ± 9.2 years, and an average of 17.2 ± 8.9 years of graduation. The female represented 63.4% of the sample, at an average age of 44.8 ± 10.4 years, and an average of 13.4 ± 7.7 years of graduation. Among the respondents, 32.2% did their postgraduate studies in Orthodontics at public institutions and 67.8%, at private institutions.

With regard to orthodontic appliances, 58.2% of orthodontists used vestibular fixed appliances, aligners, orthopedic appliances and interceptive appliances; while 5.9% used only vestibular fixed appliances and 2%, only aligners.

As for the instruments used to remove residual resins, the frequencies of usage of high speed drill, high speed drill with irrigation, low speed drill and pliers are described on Table 2. Pliers were not used to remove resin by 36.8% of respondents, while the high speed drill and high speed drill without irrigation were reported as the most frequently used instruments.

**Table 2:** Prevalence of the use of instruments to remove residual resins.

	Never n (%)	Rarely n (%)	Occasionally n (%)	Frequently n (%)	Always n (%)
High rotation drill	14 (9.21)	12 (7.89)	14 (9.21)	44 (28.9)	68 (44.7)
High rotation drill with irrigation	3 (2.1)	12 (8.7)	13 (9.4)	25 (18.2)	84 (61.3)
Low rotation drill	39 (25.4)	24 (15.6)	18 (11.7)	28 (18.3)	44 (28.7)
Pliers	56 (36.8)	37 (24.0)	24 (15.7)	17 (11.1)	19 (12.4)

There was no significant difference between men and women nor between orthodontists trained at public or private institutions, considering the frequencies of usage of high speed drill, high speed drill with irrigation, low speed drill and pliers (Pearson’s chi-square test).

Table 3 shows the type of burs used by the orthodontists. The multilaminate carbide bur was cited as the most commonly used bur for resin removal.

**Table 3:** Prevalence of the types of bur used.

	RESPONSE OPTIONS	n (%)
1	Multilaminate carbide bur	118 (82.5)
2	Multilaminate zirconia bur	36 (24.3)
3	Diamond bur	40 (27.0)
4	Aluminum oxide tip (Shofu)	63 (42.5)
5	Fiberglass tip	7 (4.7)
6	Sanding disc (Soflex Disc)	48 (32.4)
7	Rubber tip	79 (53.3)
8	Others	3 (2.0)

For 2% of the orthodontists, silicone abrasive tips, composite finishing tips and rubber polishing burs were also options to be used.

The pliers used by the responding orthodontists are presented on Table 4. The resin-removing pliers with Widia (39%), banding pliers (33,3%) and bracket-removing pliers (22.2%) were described as the most widely used for removing residual resin.

**Table 4:** Prevalence of the type of pliers used.

	RESPONSE OPTIONS	n (%)
1	Resin-removing pliers with Widia	32 (39.0)
2	Bracket-removing pliers	18 (22.2)
3	Weingart pliers	0 (0.0)
4	Tie-cutting pliers	3 (3.7)
5	Banding pliers	27 (33.3)

Orthodontists justified the use of drills and pliers to remove residual resins (Table 5): Saving time was the most frequent reason (70.6%) for using drills. For 2% of the respondents who used drills, were also justifications: places where the resin is difficult to see, cases where treatment was carried out with attachments, and the use of drills being the technique they were taught. Whereas the amount of resin to be removed and patients’ sensitivity to the use of drills were justifications for using pliers for 4.1% of respondents.

**Table 5:** Justifications for using high/low speed drills and pliers.

RESPONSE OPTIONS		DRILLS	PLIERS
		n (%)	n (%)
1	Less chance of damage to tooth enamel	40 (27.9)	36 (49.3)
2	Shorter removal time	101(70.6)	18 (24.6)
3	Less discomfort for the patient	80 (55.9)	14 (19.1)
4	Greater practicality of the technique	65 (45.4)	21 (28.7)
5	Lower cost	X	1 (1.3)
6	Less aerosol production	X	14 (19.1)
7	Others	3 (2.0)	3 (4.1)

X = not applicable.

The justifications for not using drills or pliers are displayed on Table 6. Irrigation was always used by 61.3% of the sample, and 2.1% never use it. Pain/discomfort when removing resins with high speed drill without irrigation occurred for 86% of the sample, always or frequently was reported by 42.9%, while 14% reported that never occurred.

**Table 6:** Justifications for not using high/low speed drills and pliers.

RESPONSE OPTIONS		DRILLS	PLIERS
		n (%)	n (%)
1	Increased possibility of damage to tooth enamel	20 (71.4)	31 (40.7)
2	Increased aerosol production	2 (7.1)	X
3	Longer removal time	4 (14.2)	29 (38.1)
4	Greater discomfort for the patient	7 (25.0)	42 (55.2)
5	Less practicality of the technique	4 (14.2)	35 (46.0)
6	Unfamiliarity with instruments or techniques	X	20 (26.3)
7	Others	0 (0.0)	0 (0.0)

X = not applicable.

According to orthodontists, the main reasons to remove residual resins without irrigation (Table 7) were the better visualization of the resin (100%) and less damage to enamel (40%).

**Table 7:** Justifications for not using irrigation.

	RESPONSE OPTIONS	n (%)
1	Less production of aerosol	5 (14.2)
2	Less damage to enamel	14 (40.0)
3	Shorter removal time	2 (5.7)
4	Less discomfort for the patient	0 (0.0)
5	Better visualization of the resin	35 (100)
6	Others	0 (0.0)

With regard to the techniques used to remove residual resins in orthodontic offices, 98.6% of the participants reported not using any techniques other than drills, pliers, curettes and scalpel blades. For 1.4% of the sample, using ultrasound was also an option.

The amount of residual resin was the main factor that influenced 39.2% of the professionals when deciding upon which technique to be used at any given time and considering factors prior assessed. For 3.2% of the orthodontists, other factors that ought to be taken into account when choosing a technique to use were patient sensitivity to a particular technique, patient comfort and the risk of fracturing the tooth (Table 8).

**Table 8:** Factors assessed by professionals when deciding which technique to use.

	RESPONSE OPTIONS	n (%)
1	Number of teeth with resins to be removed	10 (6.5)
2	Arch region (anterior, canine or posterior teeth)	15 (9.8)
3	Removal stage (start or finish)	27 (17.6)
4	Amount of residual resin	60 (39.2)
5	Availability of instruments at the time of removal	6 (3.9)
6	Other	5 (3.2)

## DISCUSSION

The methods for removing residual resins after the removal of orthodontic devices are cited in the literature with different recommendations,^1^ ^,^ ^8^ ^,^ ^11^ ^,^ ^13^ and controversial protocols, indicating the need for new studies.^10^

Most of the participating professionals refer to more than one type of orthodontic appliance to solve cases in their clinical practice. Despite the great advance and the gain in popularity of aligners in recent years,^14^ only 2% of the sample reported using only aligners in their offices. This result may be related to the limitations of aligners in achieving favorable anteroposterior results and in final occlusion,^15^ as well as the fact that they have been associated with greater likelihood of post-treatment relapse.^15^

The orthodontists reported that the most frequently method used to remove residual resins was the high-speed rotary instrument. Although the use of low speed has been associated with less damage to tooth enamel when compared to the use of high speed,^11^ the reason for choosing high speed revealed in this study was the shorter working time, which is an important factor reported in the literature for gaining chair time.^8^ ^,^ ^13^ ^,^ ^16^ Even so, the use of high or low speed drills causes damage to tooth enamel whether in greater or lesser quantities,^8^ which is the reason given by professionals for not using drills to remove residual resins.

The use of irrigation, reported by most professionals, is associated with greater enamel surface impairment, possibly due to the difficulty in differentiating areas with remaining resin from areas of free enamel,^11^ which is the main reason why the participants of this study do not use irrigation.

On the other hand, according to the respondents, using drills without irrigation causes more pain and discomfort, what could be explained by the greater heating of the tooth structure.^9^ The use of air-water spray is able to limit heat generation during procedures, reducing pulp temperature and generating less sensitivity.^9^

Regarding the use of drills, a protocol suggested in recent literature associated the use of the multi-laminate carbide bur for initial removal with subsequent finalization with Sof-Lex^TM^sanding discs^8^. The multi-laminate carbide bur has been extensively tested and is considered effective in removing resinous material.^8^ Its association with polishing stages of more than one step resulted in enamel surface with greater smoothness, when compared to the association with single-step polishing methods.^8^ In the present study, 82.5% of the professionals reported using the multi-laminate carbide bur to remove the resins, but only 32.4% of the sample reported using Sof-Lex^TM^ discs. The use of more than one step to remove remaining resin increases working time and the chance of damaging the enamel surface.^13^ ^,^ ^16^ This is possibly the reason why the respondents often choose to use only one instrument.

Conversely, some authors recommend using composite burs before finishing with Sof-Lex^TM^ discs,^17^ or even zirconia burs. Zirconia burs were reported by 24.3% of the participants, whereas composite burs were mentioned by only 2% of the participants. These low numbers may be related to the lack of knowledge of the professionals about the materials or to the few existing studies^10^ ^,^ ^17^ evaluating the results of the use of composite or zirconia burs on the enamel surface after the removal of residual resins in Orthodontics.

The use of pliers was justified by causing less damage to tooth enamel, while not using them was justified by greater discomfort for the patient and greater damage to the enamel. The contradiction between these findings may be related to the experience of the professionals using this technique, since the force applied incorrectly by inexperienced professionals can cause greater discomfort and more damage to tooth enamel. Another possible explanation could be the amount of remaining resin: the thinner the resin is, the less force is needed to remove it^18^, and probably with less discomfort and lower risk of damaging the enamel.

As far as the discomfort/pain for the patients caused by the use of pliers is concerned, it is known that inappropriately applied forces increase sensitivity during resin removal^19^ and also that choosing pliers that are not suitable for this purpose increase the risk of injuries to enamel structure. 

Widia resin-removing pliers were the main choice of orthodontists, possibly because they have a cutting blade designed specifically for removing residual resins.

In any case, pliers can be an alternative for those patients who report greater sensitivity to the use of drills or for areas with less residual resin, as mentioned by respondents to this study. The use of pliers in association with the use of drills is also reported in the literature^1^.

As for the techniques used, the preference for the use of drills may be related to the greater number of publications focusing on this material, making it better known and its use widely adopted by professionals.^8^ ^,^ ^10^ ^,^ ^17^ ^,^ ^19^ ^,^ ^20^ ^,^ ^21^ On the other hand, the use of ultrasound was reported as an alternative technique, but it is considered unfavorable and harmful, as it increases surface roughness and causes deep scratches on tooth structure, due to its vibratory movement^5^.

The use of high-powered lasers, particularly erbium lasers (Er:YAG and Er,Cr:YSGG), was also suggested as an alternative for removing residual resins, since they generate less pulpal heating than tungsten carbide burs.^22^ ^,^ ^23^ However, the disadvantages of lasers are longer working time and higher investment costs, when compared to drills,^23^ which may be why they were not mentioned as techniques in the present study.

The main factor influencing the choice of residual resin removal technique was the amount of remaining resin, what could be explained by the decision to use faster techniques when the amount of residual material is greater; or techniques less aggressive for enamel when the resin layer is less thick.

The results of this study suggest that orthodontists prioritize working time when choosing resin removal methods, rather than the lowest risk of damage to tooth enamel. This is contrary to excellent professional practice, which should prioritize the choice of safe methods with the best results. The devaluation of compensation and the precariousness of dental work^24^ may be the possible reasons for these findings, highlighting the importance of future studies that consider the relationship between costs and effectiveness in Orthodontics. The publication of a recent systematic review suggested that the economic discussion in Orthodontics is scarce and limited, requiring new studies on the subject.^25^

This study sought to present the most common practices involving the residual resin removal stage. However, the result obtained after removing the adhesive residue from dental enamel should be considered as more important than the techniques used, since the smaller the area involved by the clinical procedures carried out and/or the less rough the enamel surface becomes, the less plaque will be retained.^26^ ^,^ ^27^

There are a lot of residual resin removal techniques; however, it is known that the answers collected do not always reflect day-to-day practices, and a possible solution is to associate the questionnaire with observation visits to the offices - even so, temporary changes in clinical practice can be made during data collection period.

Nonetheless, the collected data makes it possible to discuss the most commonly used practices in relation to those that are most recommended, as well as highlighting possible economic questions related to the choice of these practices. Further studies on this subject would allow the results to be compared, helping to draw up protocols that consider sustainable costs and reasonable clinical time, so that they can be applied to the daily routine of orthodontic clinics.

## CONCLUSION

The method for removing residual resins most used by the professionals was the multi-laminate carbide bur at high speed with irrigation, justified by the shorter working time.
